# The insight through the current immunotherapeutic guidelines for infectious diseases

**DOI:** 10.1097/JS9.0000000000000152

**Published:** 2023-01-27

**Authors:** Olivier Uwishema, Goodluck Nchasi, Glorian Goodluck Nnko, Elias Mtawala, Deusdedith Boniphace Bulimbe, Ghalib Haidari Kassim, James Mushi, Abubakar Nazir, Criselle Angeline Peñamante

**Affiliations:** a Department of Research and Education, Oli Health Magazine Organization, Kigali, Rwanda; b Clinton Global Initiative University, New York, New York, USA; c Faculty of Medicine, Karadeniz Technical University, Trabzon, Turkey; d Catholic University of Health and Allied Science, Mwanza; e University of Dodoma, Dodoma; f Muhimbili University of Health and Allied Sciences, Dar es Salaam, Tanzania; g King Edward Medical University, Lahore, Pakistan; h Department of Clinical Epidemiology, Faculty of Medicine and Surgery; i Department of Psychology, College of Science, University of Santo Tomas, Manila, Philippines

Highlights

- The recent rise in antimicrobial resistance has increased the burden of infectious diseases caused by bacteria, parasites, and viruses. The development of immunotherapy provides new treatment options in the fight against infectious diseases.
- host-directed treatment options are now becoming viable adjuncts to standard antimicrobial treatment, despite having some toxicities such as dermatologic, gastrointestinal, thyroid, pituitary, neurologic, renal, ocular, cardiovascular, and hematologic toxicit.
- Improving the management of infectious diseases requires the development and use of immunological therapy. Some of our suggestions for using immune treatment to lessen the impact of viral diseases include. Increased vaccine production and supply should be emphasized.

*Dear Editor,*

Coronavirus disease 2019 (COVID-19) has opened opportunities for immunotherapy as an effective option in treating emerging diseases. Immunotherapy act against pathogens and infected host cells by manipulating immune system components to target and eradicate them. Immunotherapy has been mostly associated with cancer treatment but initiatives are made to curative different diseases such as malaria, zika virus, HIV, tuberculosis, and COVID-19^1^. Mutations of viruses such as Epstein–Barr virus, cytomegalovirus, and HIV have led to emergency and re-emergence of viral infections, making effective therapy difficult^2^. Hence, the use of antibodies, primarily monoclonal antibodies and multispecific antibodies, has been one of the strategies of immunotherapy in bringing about activation of the immune system^3^. For one, the hepatitis B virus is viral inactivating the innate immune system, rendering it weak. However, if the innate immunity is effective, hepatitis B virus replication may be suppressed^4^. Consequently, the role of immunotherapy in activation of the innate immunity can be acknowledged. Studies have shown that chronic viral infections play an important role in T-cell exhaustion responsible for mediating bodily cellular immunity^5^. Once the T-cell immunity arm of our body’s immune system is altered, the body automatically becomes a reservoir of chronic infections. Hence, the focus should be on reversing the exhausted state of the T cells^6^. The existence of pathogen-associated molecular proteins in bacterial pathogens has paved way to the development of strategies to activate various receptor modules in adjuvant immunotherapy to treat bacterial infections. Polybacterial immunostimulants, such as *Respivax,* have been tested. Clinical data has shown it to be a prospective treatment for respiratory infections in complex treatment in some immunodeficiency states such as AIDS^7^. There is also a role of synthetic immunobiotics in the adjuvant treatment of bacterial infections. Fusogenic silicon nanoparticles were found effective in improving the therapeutic outcome in mice infected with methicillin-resistant *Staphylococcus aureus* and *Pseudomonas aeruginosa* 8. Synthetic polymeric scaffolds with vancomycin/fluorescein polyvalent polymers have been tested *in vitro* to be effective against *S. aureus*, *Staphylococcus epidermidis*, and *Streptococcus pneumoniae* 8. The most tested vaccine for the prevention of *Plasmodium falciparum* is RTS,S/ASO1, which contains a virus-like particle – the terminal domain of *P. falciparum* circumsporozoite protein combined with hepatitis B virus surface antigen being formulated with potent liposomal adjuvant system ASO1. Although the vaccine has proven efficacy against clinical malaria in phase III trials, protection is partial as subsequent declines ensue with time. Immunotherapy is linked to the following toxicities: dermatologic, gastrointestinal, hepatitis, thyroid, pituitary, neurological, renal, ocular, cardiovascular, and hematological toxicities. In the antimicrobial resistance era, immunotherapy is important to fight against emerging infectious diseases. Some of our suggestions for using immune treatment to lessen the impact of viral diseases include. Increased vaccine production and supply should be emphasized. Vaccination is essential in improving immunity against infectious diseases such as polio, measles, and pneumonia^9^. According to the WHO, ∼1.5 million children die under the age of 5 of vaccine-preventable diseases annually^9^. The tolerability, safety, and effectiveness of currently existing vaccines necessitate improvement. This is crucial since vaccine-preventable diseases like measles, mumps, and rubella are no longer present due to the rapid mutation of the majority of infectious pathogens. The development of nanotechnology which improves immune system functionality is of paramount importance, especially for HIV and tuberculosis where vaccine strategies hardly generate protective immunity in the population^10^. The use of self-assembled protein nanoparticles has proven essential in the development of the malaria vaccine since it provides a useful platform for antigen delivery. The burden of infectious diseases has increased as a result of the development of antibiotic resistance in the second half of the 20th century. For the efficient treatment of infectious diseases brought on by bacteria, parasites, and viruses, improved therapeutic modalities like immune therapy must be developed and used. Despite adverse drug reactions associated with immunotherapies, multidisciplinary research partnerships must be supported to create better vaccines, nanotechnology, and other therapeutic approaches.

## Ethical approval

Not applicable.

## Sources of funding

Not applicable.

## Authors’ contribution

O.U. contributed in the conceptualization, project administration, writing − review and designing. All authors were involved in the writing of manuscript, data collection and assembly, and final approval of manuscript.

## Conflicts of interest disclosure

The authors declare that they have no financial conflict of interest with regard to the content of this report.

## Research registration unique identifying number (UIN)

Not applicable.

## Guarantor

Abubakar Nazir.
